# Not Aging but Calorie Restriction Strongly Affects Protein Oxidation in Heart and Brain Mitochondria

**DOI:** 10.1111/acel.70339

**Published:** 2025-12-28

**Authors:** Shipan Fan, Carina Ramallo‐Guevara, Monika Frenzel, Shuichi Yanai, Sataro Goto, Michiru D. Sugawa, Norbert A. Dencher, Ansgar Poetsch

**Affiliations:** ^1^ School of Basic Medical Sciences, Institute of Biomedical Innovation, Jiangxi Medical College Nanchang University Nanchang Jiangxi China; ^2^ Plant Biochemistry, Faculty of Biology & Biotechnology Ruhr University Bochum Bochum Germany; ^3^ Physical Biochemistry, Department of Chemistry Technical University of Darmstadt Darmstadt Germany; ^4^ Tokyo Metropolitan Institute for Geriatrics and Gerontology Tokyo Japan; ^5^ Charité Universitätsmedizin Berlin Berlin Germany

**Keywords:** aging, calorie restriction, mitochondria, oxidative modification

## Abstract

Aging is an inevitable consequence for all organisms. According to the mitochondrial free radical theory of aging (MFRTA), reactive oxygen species (ROS), which are predominantly generated in mitochondria, are assumed to play a key role. Calorie restriction (CR) delays aging by improving mitochondrial function; however, the molecular mechanisms underlying the effects of ROS and CR on mitochondria remain poorly understood. Oxidative protein modifications in mitochondrial proteins from the heart and cerebrum of young (6.5 months) and old (27 months) rats were quantified and the effects of short‐term and lifelong CR interventions were investigated. Mass spectrometry was leveraged to achieve an unbiased and comprehensive analysis of various types of oxidative postranslational modifications (oxPTMs). Contrary to the MFRTA, aging did not cause significant increases in mitochondrial protein oxidation in the heart and cerebrum. CR markedly diminished the overall level of oxPTMs in the heart, particularly in transmembrane proteins. Similarly, the level of oxidative modification of transmembrane proteins in cerebrum was reduced by CR, whereas it perplexingly increased in mitochondrial proteins. The absolute level of oxidized mitochondrial protein was always higher in the heart than in the cerebrum under all conditions. Carbonylation, a prevalent marker of protein oxidation and aging, increased in the heart with age and was notably reduced by CR. However, this trend was not consistent in cerebrum or for some other types of oxPTMs. Therefore, protein oxidation in the heart and cerebrum exhibits distinct responses to chronological aging and dietary interventions, with the latter exerting a stronger influence.

## Graphical abstract

Our comprehensive proteomic analysis reveals that calorie restriction (CR), not aging, is the primary driver of mitochondrial protein oxidation, with striking tissue‐specific effects. While CR reduces oxidation in the heart, it exerts a dual and opposing role within the cerebrum.

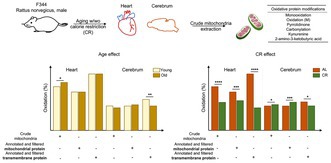

## 1 Introduction

Aging is a progressive, complex, and biologically inevitable process. Many aspects of the aging process remain enigmatic, and the relevance of the myriad of existing theories and contributing factors remains a subject of active research and debate. The Mitochondrial Free Radical Theory of Aging (MFRTA), which Denham Harman proposed in 1956, has grown to be one of the most researched and prominent hypotheses in the field of aging studies. It claims reactive oxygen species (ROS), produced in mitochondria, drive the aging process by accumulating oxidative damage (Harman 1956; Miquel et al. 1980; Shigenaga et al. 1994). Free radicals can be converted to other, less reactive and longer‐lived ROS (e.g., H_2_O_2_). Both short‐lived free radicals and longer‐lived ROS can damage biomolecules (Aruoma 1998; Doonan et al. 2008; Kozlov et al. 2024; Zhang et al. 2009). On the other hand, free radicals, most of which are ROS, participate in signaling pathways and are involved in vital physiological activities such as immune responses and cell proliferation (Holmstrom and Finkel 2014; Rhee 2006; Wang and Hekimi 2015). While a plethora of past studies support the MFRTA theory (Ishii et al. 2002; Schriner et al. 2005), in recent years it has been challenged based on newer research that uncovered an inconclusive and complex relationship between mitochondrial ROS production and aging (Ristow and Schmeisser 2011). Interventions in increasing or decreasing antioxidant defenses, such as supplementing antioxidants and knocking out the antioxidant enzymes, have not always extended lifespan (Bjelakovic et al. 2007; Lin et al. 2009; Sanz et al. 2006; Sumien et al. 2003) or even accelerated aging. Moreover, long‐lived animals can maintain a long lifespan despite high levels of free radical production (Jones et al. 2014; Omotoso et al. 2021). Given that age‐related changes are more pronounced in postmitotic tissues, such as the brain, heart, and skeletal muscle, these tissues are the objects of most aging studies (Boengler et al. 2017; Kwong and Sohal 2000; Tuttle et al. 2020).

Calorie restriction (CR) increases lifespan (Goto 2019) and alleviates age‐related pathologies in various species. It is generally assumed that CR exerts these benefits partly by reducing mitochondrial ROS production, thereby preventing oxidative damage in different tissues (Escobar et al. 2019; Guarente 2008; Sohal et al. 1994; Strickland et al. 2019). However, the specific mechanisms involved in the relationship between CR, the production of ROS levels, and health span extension are not fully understood (Dani et al. 2010). Considering the high energy demands of organs such as the heart and brain, these organs rely heavily on mitochondrial function, which is closely associated with age‐related pathologies. For instance, the heart must constantly pump blood to maintain circulation, and the brain, which constitutes 2% of the total body weight but consumes more than 20% of the body's total energy, requires a significant amount of energy to support neuronal activity and information processing (De Nicolo et al. 2023; Trigo et al. 2023). It is worth noting that the cerebrum represents a particularly valuable model for the study of neurodegenerative disorders and brain development because of its complexity and significance in cognitive function.

The deterioration of mitochondrial function is regarded as an inevitable consequence of the aging process and a contributing factor to the development of age‐related diseases (Amorim et al. 2022; Sun et al. 2016). ROS, the main byproduct generated by the electron transport chain in the inner mitochondrial membrane, can oxidize biomolecules such as DNA, lipids, and proteins. Among these, oxidized proteins have been linked to inevitable outcomes such as irreversible alterations of protein structure and functionality in cellular activity, and they contribute to age‐related diseases (Cross et al. 1987). The types of protein oxidation are structurally complex. Reversible modifications are relevant to physiological processes and are involved in signaling mechanisms. In contrast, irreversible modifications may contribute to pathological changes and to the onset of various diseases (Kehm et al. 2021).

The landscape of protein modifications induced by ROS is vast and analytically challenging. The modifications commonly investigated are carbonylation, oxidation of methionine and cysteine residues, nitration, and the formation of advanced glycation end products (AGEs) (Demasi et al. 2021; Kim et al. 2015; Moldogazieva et al. 2019). Although an arsenal of antibodies has been developed (Ladouce et al. 2023) to detect specific oxidative PTMs (oxPTMs), these methods are often hampered by cross‐reactivity and impracticality of simultaneously investigating a wide range of modifications or proteins. In recent years, mass spectrometry has evolved to be a superior alternative for the analysis of oxPTMs. Sophisticated workflows have been developed that usually involve enrichment of low‐abundance oxPTMs. Examples include oxiTMT for cysteine redoxome (Shakir et al. 2017), ^16^O/^18^O H_2_O_2_‐labeling for methionine oxidation (Liu et al. 2013), and biotin hydrazide for protein carbonylation (Soreghan et al. 2003). Targeting a specific modification provides high sensitivity and quantitative accuracy. However, the tradeoff is a restricted view of the PTM landscape. This limitation is problematic when the relevance of the investigated PTM for aging is initially unclear. In this case, an unrestricted, yet less sensitive, investigation of oxPTMs should be more appropriate and has been previously employed by us (Ramallo Guevara et al. 2016) and others (Rykaer et al. 2017).

The objective of our work was to gain a comprehensive understanding of the oxidative protein changes occurring in mitochondria during the aging process and the consequences of CR. We used untargeted and unbiased proteome analysis to detect and quantify prominent types of oxPTMs. This unbiased strategy was chosen because no universal technology exists to enrich all oxPTMs, and it allows for a global comparison (Verrastro et al. 2015). We individually analyzed (a) proteins with at least one predicted transmembrane helix (transmembrane proteins) and (b) proteins annotated as mitochondrial (mitochondrial proteins). The key findings of our study were that aging only mildly affected protein oxidation, whereas CR markedly diminished it in the heart mitochondria. This effect was tissue‐specific: in the cerebrum, CR decreased protein oxidation only for transmembrane proteins, whereas, for annotated mitochondrial proteins, protein oxidation increased with CR. In conclusion, the present study provides novel insights into the mechanisms underlying aging and its tissue heterogeneity, modulation due to CR, and has relevance for future treatment of cardiovascular and neurodegenerative disorders.

## 2 Results

**FIGURE 1:**
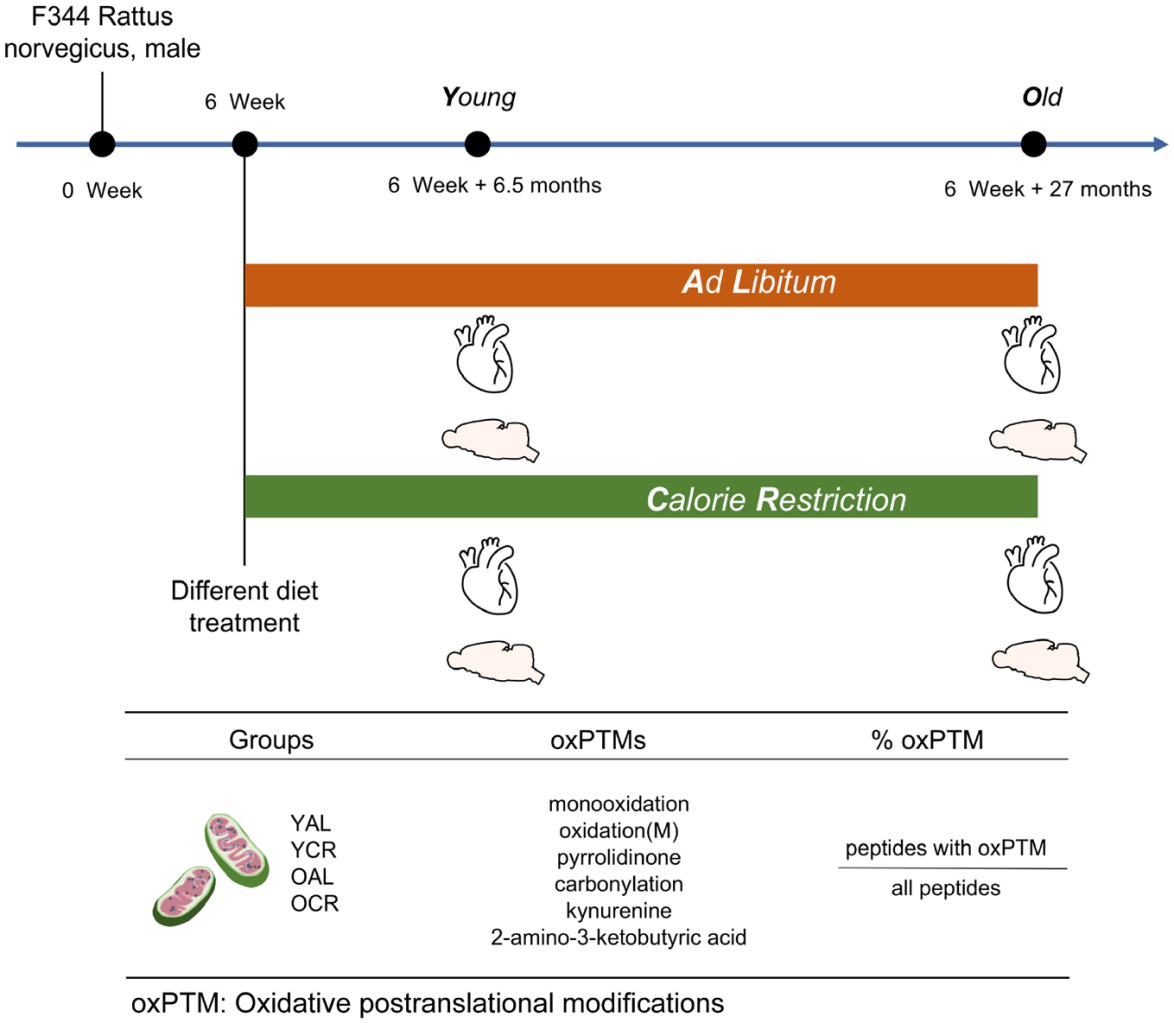
The experimental design and methodology for analyzing oxidative postranslational modifications (oxPTMs).

It is important to note that prior to this extensive proteomic investigation, an OxyBlot analysis was conducted on mitochondrial proteins extracted from the cortex of young and aged rats. This initial experiment revealed a higher level of global protein carbonylation in young than aged rats, which contrasted with the predictions of the MFRTA (Figure S1). This intriguing observation underscored the limitations imposed by PTM‐constrained, antibody‐based methodologies and the intricate nature of oxidative protein modifications during the aging process. Therefore, to achieve an unbiased and comprehensive understanding, an untargeted proteomics approach was employed to quantify a broad spectrum of oxPTMs in response to both aging and CR. Rats were fed ad libitum (AL) or calorie restricted (CR) and samples obtained from young (Y), and old animals (O). This initial experiment used samples from the full cohort of animals (YAL, *n* = 5; YCR, *n* = 5; OAL, *n* = 6; OCR, *n* = 6). The 50% survival of CR rats was at 1097 days as compared to 855 days of AL fed rats, being 28% longer lived in the former (Figure S2). The alternate‐day fasting protocol induced a significant reduction in body weight in the CR groups (YCR, OCR) compared to their age‐matched AL controls (Figure S3). These physiological (*n* = 5–6 per group) and survival analyses (*n* = 40 for CR; *n* = 21 for AL) were performed on the complete set of animals. Subsequent to this, the normalization of organ weights to body weight revealed that CR increased the relative ratio of the cerebrum, but not the heart, with the parallel reduction in absolute heart weight and total body weight suggesting a tight coupling between them (Figure S4). For the subsequent in‐depth mitochondrial proteomic and oxPTM analyses, a representative subset of three animals from each of the four groups was used (resulting in *n* = 3 per group for all proteomics data). Crude mitochondrial fractions from the cerebrum and heart were used to potentially retain valuable biological information from various forms of mitochondria. The term “oxidation” is used there as a broad category encompassing all abundant oxPTMs listed below. These included monooxidation (mass shift: +15.9949 Da, +1O on lysine, aspartic acid, histidine, leucine, phenylalanine, proline, tryptophan), oxidation(M) (mass shift: +15.9949 Da, +1O on methionine), pyrrolidinone (mass shift: −30.0106 Da, −2H−1C−1O on proline), carbonylation (mass shift: +13.9793 Da, −2H+1O on arginine, leucine, proline), kynurenine (mass shift: +3.9949 Da, −1C+1O on tryptophan), and 2‐amino‐3‐ketobutyric acid (mass shift: −2.1056 Da, −2H on threonine), and were identified by their respective mass shift as variable modification sites for data processing with MaxQuant (Figure 1, Table S1). The protein list was filtered for mitochondrial proteins or proteins with transmembrane (TM) domains according to the UniProt database. This large‐scale iTRAQ study quantified a total of 2105 proteins from cerebrum and 858 from heart in tissue‐specific crude mitochondrial proteomes. Filtering for mitochondrial proteins was done to allow comparison of the mitochondrial proteome from both tissues, given that the mitochondrial fraction substantially differed in identified co‐purified proteins between the heart and cerebrum (Table S2). A focused view of TM proteins accounted for their unique physicochemical features (amino acid composition, localization, hydrophobicity etc.) and crucial role in energy conversion, material transport, and signal transduction (Muller et al. 2008; Pfanner et al. 2019). Furthermore, integral respiratory chain complexes I and III are major sources of ROS that may inflict oxidation of nearby proteins. For quantification, the number of peptides with oxPTMs was counted and normalized to the total number of identified peptides to calculate the ratio for each sample. The data were analyzed for mitochondrial protein oxidation, transmembrane protein oxidation, individual oxPTMs, and any specific contribution of aging, diet, and tissue. In the present study, analysis of total oxPTMs (i.e., without filtering for mitochondrial or TM proteins) across tissues revealed a distinct baseline susceptibility to oxidative modifications. Proteins in the heart exhibited significantly higher levels of total oxPTMs compared to those in the cerebrum, accounting for approximately 35% compared to 21% of all identified proteins, respectively (Figure S5), indicating a greater inherent susceptibility of heart proteins to ROS‐induced modifications. Notably, aging alone did not result in a significant alteration of total oxPTM levels in the cerebrum under either dietary condition, whereas a trend of significant increase was observed in the heart with age under both dietary modes.

### 2.1 Not Aging, but CR Substantially Affected Protein Oxidation of Heart Mitochondrial Proteins

**FIGURE 2:**
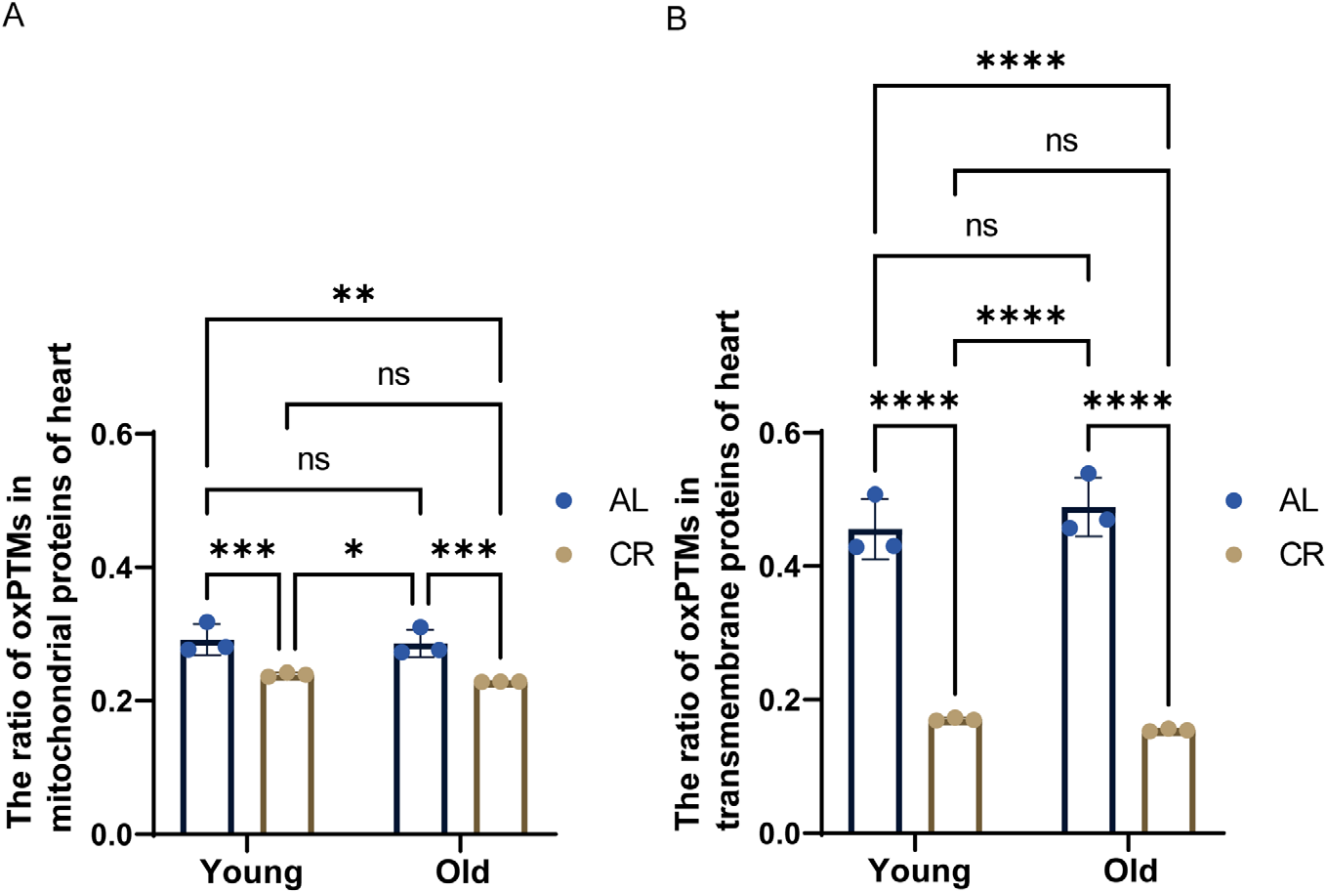
CR significantly decreased protein oxidation in the heart while aging had no effect. The ratio of oxPTMs in mitochondrial protein (A) and in transmembrane protein (B) in heart. *n* = 3, two‐way ANOVA. The ratios were calculated as the number of identified peptides with oxPTM normalized to the total number of identified peptides. AL, ad libitum; CR, calorie restriction; Y, 6.5 months; O, 27 months; oxPTMs, oxidative postranslational modifications. Histograms represent the mean ± SD. *p < 0.05, **p < 0.01, ***p < 0.001, ****p < 0.0001, ns, not significant by Student *t*‐test.

The extent of protein oxidation of annotated mitochondrial proteins derived from the heart was evaluated. While aging did not affect protein oxidation, there was a pronounced effect of CR. The significant effect of CR on oxPTMs, around 19% reduction, was demonstrated for mitochondrial proteins in both the comparison of YCR to YAL and OCR to OAL (Figure 2A). For membrane‐integral proteins, aging did not affect the level of total oxPTMs in the heart; however, the CR intervention resulted in a striking decrease in the total oxPTM ratio, exceeding 50% (Figure 2B). Furthermore, the oxidative modification level of membrane‐integral proteins was even higher than that of mitochondrial proteins, as well as the total oxPTM level (Figure 2, Figure S5A). The finding that membrane proteins are more susceptible to oxidative modification has been described for humans, too (Granold et al. 2015). The longer half‐life of heart mitochondrial proteins should increase the likelihood of accumulation of oxidative damage (Dai et al. 2014) relative to other cell compartments, although this could not be investigated in our study because only the mitochondrial fraction was analyzed. Nonetheless, consistent with the previous study (Dai et al. 2014) for short‐term CR, CR decreased the oxPTM levels of mitochondrial and transmembrane proteins in the hearts of young and old animals. Moreover, oxPTMs in transmembrane proteins exhibited a more sensitive response to CR than mitochondrial proteins.

### 2.2 CR Differently Affected the Oxidation of Mitochondrial and Transmembrane Proteins in the Cerebrum

**FIGURE 3:**
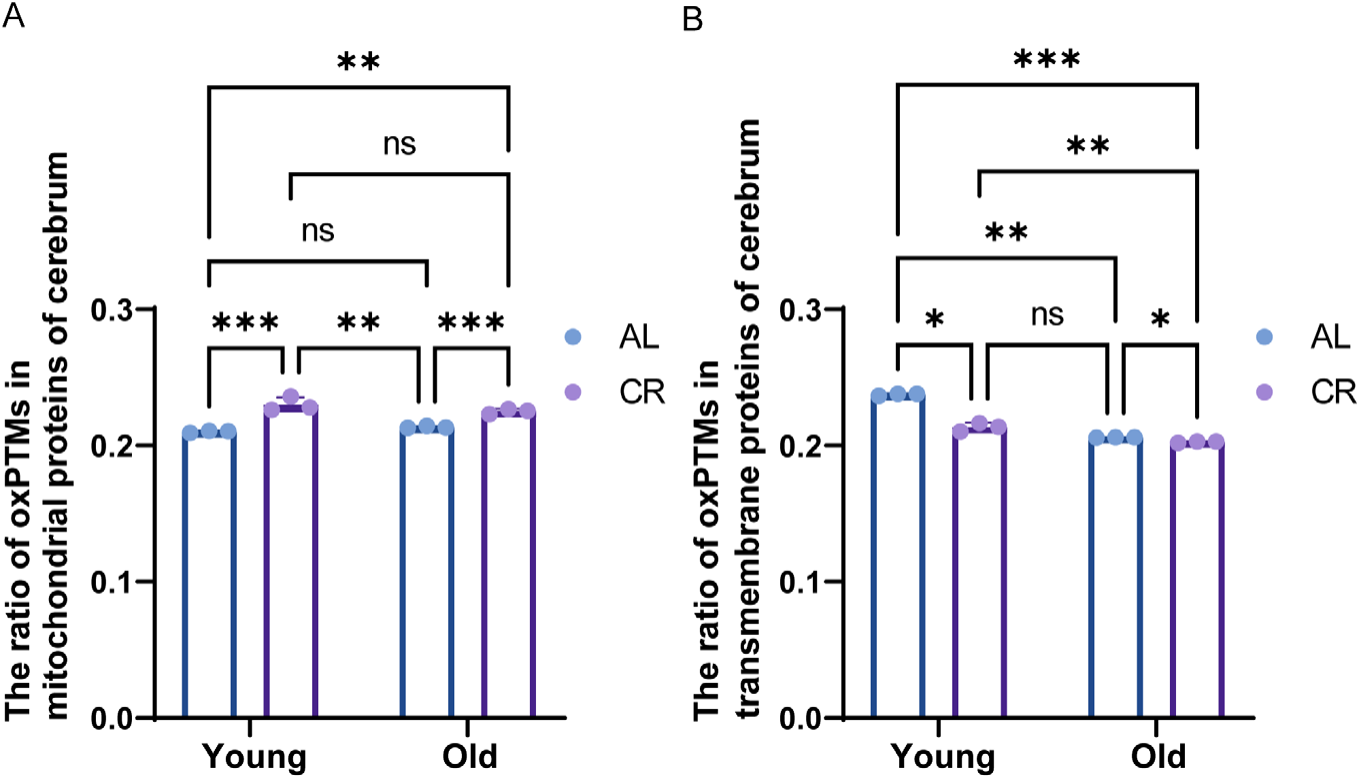
Aging and CR decrease oxPTMs in transmembrane protein but CR increases mitochondrial protein oxPTMs. The ratio of oxPTMs in mitochondrial protein (A) and in transmembrane protein (B) in cerebrum. *n* = 3, two‐way ANOVA. The ratios were calculated as the number of identified peptides with oxPTM normalized to the total number of identified peptides. AL, ad libitum, CR, calorie restriction; Y, 6.5 months; O, 27 months; oxPTMs, oxidative postranslational modifications. Histograms represent the mean ± SD. *p < 0.05, **p < 0.01, ***p < 0.001, ****p < 0.0001, ns, not significant by Student *t*‐test.

Energetic requirements of the brain severely rely on mitochondrial functionality. Moreover, the brain displays pronounced heterogeneity in protein turnover and its marked decrease at higher age, with cells and regions of low turnover thus more susceptible to accumulation of oxidized proteins (Fernandez‐Sanz et al. 2014; Rao et al. 2024). Therefore, the brain is a promising object in aging research that aims to dissect the role of ROS in the aging process. Regarding the influence of aging, no significant alterations were observed in OAL compared with YAL; also the comparison of OCR with YCR, which indicated that the mitochondrial protein oxidation level was not affected by the aging process (Figure 3A). However, a more detailed examination of oxPTM alterations in transmembrane proteins revealed a notable decline in the level of transmembrane protein oxPTMs in the cerebrum with age, which further decreased under the CR status. Among these alterations, the levels of oxPTMs in YCR exhibited more pronounced changes than those in OCR, indicating that transmembrane proteins may decline at a higher age (Figure 3B). The functional relevance of the proteomic changes was assessed by measuring mitochondrial membrane “fluidity” (Heyn, Blume, et al. 1981; Heyn, Cherry, and Dencher 1981). We found that despite the overall reduction in transmembrane protein oxidation, aging under AL diet significantly decreased membrane fluidity (increased anisotropy), an indicator of membrane perturbance. Long‐term CR effectively mitigated this age‐related rigidification (Figure S6). In contrast to the results observed for transmembrane proteins, the degree of oxPTMs in mitochondrial proteins was significantly upregulated when comparing YCR to YAL or OCR to OAL. Moreover, a larger variance was observed in the CR‐treated samples for both YCR and OCR compared to AL (Figure 3A). Although this variance may have a biological foundation (interindividual variability in responses to CR), underlying technical reasons cannot be excluded (e.g., sample preparation or the quantification of low‐abundance proteins).

### 2.3 Heart Displays No Uniform Trend in Protein Oxidation(M) and Monooxidation

It is essential to investigate the amount and changes of individual oxPTMs to infer their relevance to aging and CR in the heart. As described above, the overall oxPTMs of mitochondrial proteins and TM proteins did not significantly change with age in the heart under AL, to which monooxidation and oxidation(M) contributed > 90% (Figure S7A,B, Table 1). However, individual oxPTMs may deviate from the overall trend, which has possible implications for the aging process. Moreover, it seems reasonable to assume that changes in the abundance of highly abundant oxPTMs are more relevant than the less abundant. The carbonylation level in proteins is widely regarded as a marker of oxidative damage to proteins (Akagawa 2021). This was also confirmed in our study, which identified the ratio of annotated mitochondrial and TM peptides in Table S3.

**TABLE 1:** Alterations in each oxidative PTM in the heart during aging under AL and CR diet.

Tissue	Diet	Age	PTMs	Mitochondrial proteins			Transmembrane proteins		
				Average ratio (%)	Diet effect	Age effect	Average ratio (%)	Diet effect	Age effect
Heart	AL	Y	2‐amino‐3‐ketobutyric acid	0.18			0.08		
			Carbonylation	0.45			0.62		
			Kynurenine	0.00			0.23		
			Oxidation(M)	16.54			20.61		
			Pyrrolidinone	0.07			0.08		
			Monooxidation	11.94			23.93		
		O	2‐amino‐3‐ketobutyric acid	0.17		ns	0.06		ns
			Carbonylation	0.51		ns	0.67		ns
			Kynurenine	0.02		**↑**	0.00		**↓**
			Oxidation(M)	15.24		ns	20.78		ns
			Pyrrolidinone	0.02		**↓**	0.00		**↓**
			Monooxidation	12.67		ns	27.37		ns
	CR	Y	2‐amino‐3‐ketobutyric acid	0.02	**↓**		0.26	**↑**	
			Carbonylation	0.09	**↓**		0.85	**↑**	
			Kynurenine	0.00	—		0.09	**↓**	
			Oxidation(M)	14.53	**↓**		8.13	**↓**	
			Pyrrolidinone	0.17	**↑**		0.04	**↓**	
			Monooxidation	9.11	**↓**		7.70	**↓**	
		O	2‐amino‐3‐ketobutyric acid	0.03	**↓**	**↑**	0.00	ns	**↓**
			Carbonylation	0.01	**↓**	**↓**	0.18	**↓**	**↓**
			Kynurenine	0.00	**↓**	—	0.00	—	**↓**
			Oxidation(M)	15.08	ns	**↑**	8.96	**↓**	**↑**
			Pyrrolidinone	0.04	**↑**	**↓**	0.21	**↑**	**↑**
			Monooxidation	7.71	**↓**	**↓**	6.11	**↓**	**↓**

*Note:* The up arrow: significantly increased; The down arrow: significantly decreased; −: not available; Y: 6.5 months, O: 27 months. The most frequent oxPTMs were not significantly affected by age in the AL group.

Abbreviations: AL, ad libitum; CR, calorie restriction; ns, not significant.

**FIGURE 4:**
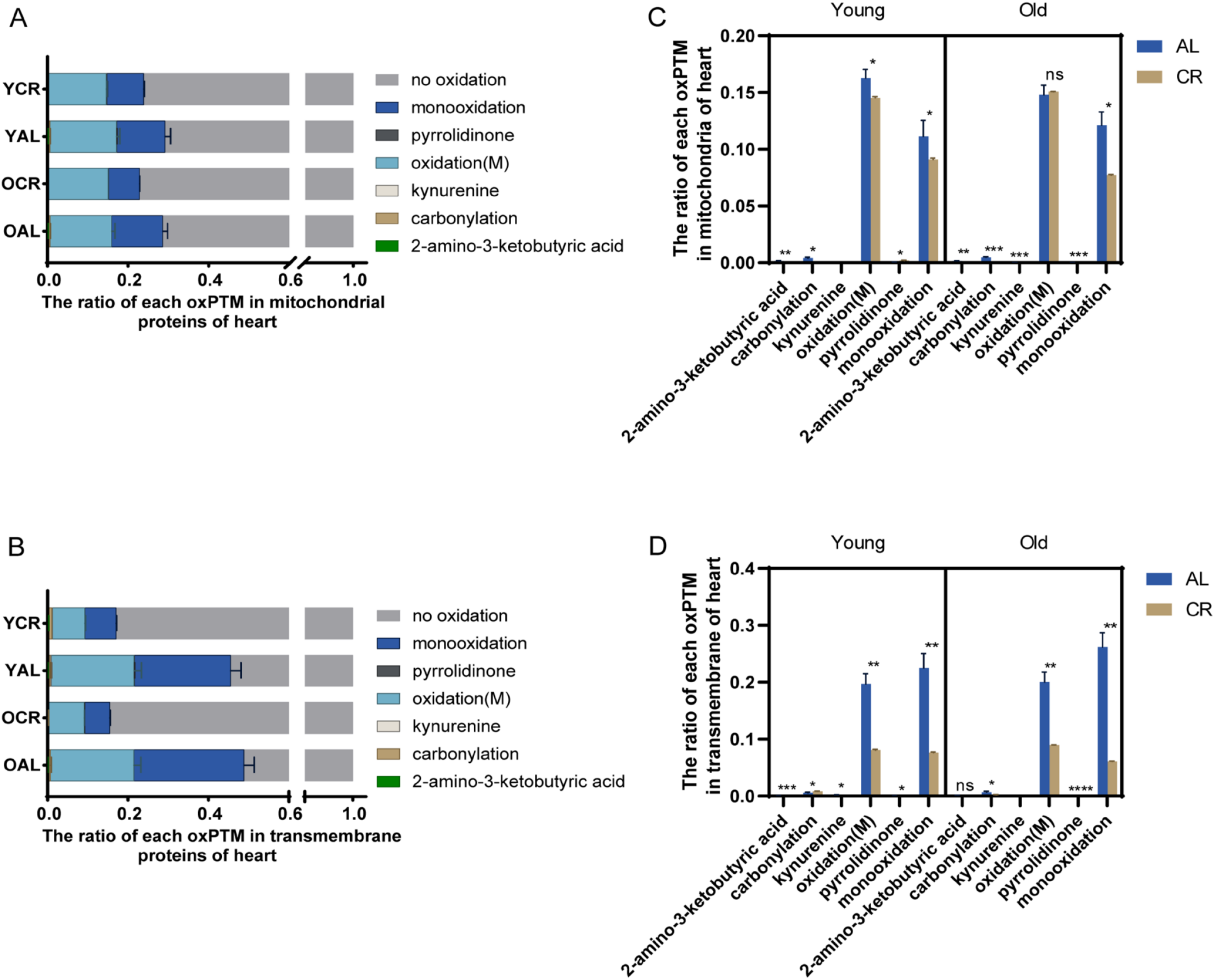
Oxidation(M) and monooxidation were most frequently observed and affected by age and diet in heart. The bar chart illustrates the ratio of each identified oxPTM in mitochondrial protein (A) and each identified oxPTM in transmembrane protein (B) in each animal (*n* = 3). The ratios represent the number of identified peptides with oxPTM normalized to all identified peptides in the sample. Error bars indicate the standard deviation among biological replicates (*n* = 3) per oxidative modification site. The effect of CR on each oxPTM in mitochondrial protein (C) and transmembrane protein (D) of the heart under short‐ and long‐term conditions. *n* = 3, unpaired *t*‐test. *p < 0.05, **p < 0.01, ***p < 0.001, ****p < 0.0001, ns, not significant by Student *t*‐test.

Among the mitochondrial proteins, the relative abundances of oxPTMs, comprising 2‐amino‐3‐ketobutyric acid, kynurenine, and pyrrolidinone were markedly lower than that of monooxidation, oxidation(M), and carbonylation (Figure 4A,C, Table 1). Interestingly, pyrrolidinone levels were reduced in both the AL and CR groups with the progression of aging. The alterations between the YCR and OCR groups presented various changes that differed from those observed between the YAL and OAL groups. Among these, the effects of age on the OCR group were notable compared with the YCR group, with an increase in 2‐amino‐3‐ketobutyric acid and oxidation(M), whereas the levels of carbonylation, monooxidation, and pyrrolidinone exhibited a substantial decline (Figure S7A, Table 1). A comparison between YAL and YCR, along with OAL and OCR, revealed a reduction in the proportions of 2‐amino‐3‐ketobutyric acid, carbonylation, and monooxidation due to CR administration, except for pyrrolidinone, which exhibited a significant increase. Moreover, oxidation(M) decreased in YCR compared to YAL. However, no significant alterations were observed in the long‐term CR mode (OAL/OCR). In contrast, kynurenine levels were decreased only in the OCR group. Notably, the increase in pyrrolidinone levels remained elevated after both short‐ and long‐term CR treatment (Figure 4C, Table 1). The results demonstrate that CR intervention exerts a mitigating effect on most oxidative modification in mitochondrial proteins. Furthermore, mitochondrial proteins are more profoundly affected by age in response to CR than in response to the AL diet. TM proteins may be more strongly affected by their proximity to sources of reactive oxygen, the higher frequency of methionine (Gomez‐Tamayo et al. 2016), and their overall lower turnover in the heart, as reported (Geldon et al. 2021). To verify this hypothesis, variations in each oxPTM of TM proteins under different conditions were compared, and it was found that the majority of detected oxidative modifications in each group were still accounted for by two types of modification: oxidation(M) and monooxidation (Figure 4B,D). With regard to the impact of age in AL regime, the rare modifications of kynurenine and pyrrolidinone were not identified for OAL, hence were significantly reduced in comparison to YAL (Figure S7B, Table 1). In comparison with the YCR group, the ratios of 2‐amino‐3‐ketobutyric acid, carbonylation, kynurenine, and monooxidation decreased with age in the OCR group. Additionally, there was a considerable rise in the frequent methionine oxidation and pyrrolidinone in OCR when compared to YCR (Table 1, Figure S7B). Considering the effect of dietary restriction, a reduction in the levels of oxPTMs was observed in YCR compared to YAL including kynurenine, oxidation(M), pyrrolidinone, and monooxidation. In contrast, 2‐amino‐3‐ketobutyric acid and carbonylation levels were elevated. Nevertheless, OCR had the opposite effect of reducing carbonylation and increasing pyrrolidinone (Figure 4D, Table 1). It is worth noting that the relative alteration of oxidation(M) and monooxidation in transmembrane proteins was about 61% (from 21% to 8%) and 68% (from 24% to 8%), respectively, with a decrease in YAL versus YCR; 57% (from 21% to 9%) and 78% (from 27% to 6%) decrease in OAL versus OCR, respectively. The effect of CR on protein oxidation was much greater for transmembrane proteins than for mitochondrial proteins, with monooxidation being particularly prominent (Table 1).

In summary, in both mitochondrial and TM proteins, none of the major oxidative protein modifications increased at high age, whereas they decreased under CR and changed mostly in TM proteins. To further delineate specific oxidative damage targets, we analyzed tissue‐specific methionine oxidation at the protein level. This revealed that identical proteins can be oxidized at distinct methionine residues in the heart compared to the cerebrum, and these sites exhibited divergent regulation by aging and CR (Figure S8). For instance, while Atp5b‐M272 and Ndufs1‐M297 were oxidized in both tissues, their responses differed markedly. In the heart, oxidation at these sites was consistently elevated by CR. Conversely, in the cerebrum, Ndufs1‐M297 oxidation increased with age but remained elevated with YCR, whereas Atp5b‐M272 oxidation exclusively increased in the YCR group.

### 2.4 In Cerebrum Individual oxPTMs Were Hardly Affected by Aging but by Diet

**FIGURE 5:**
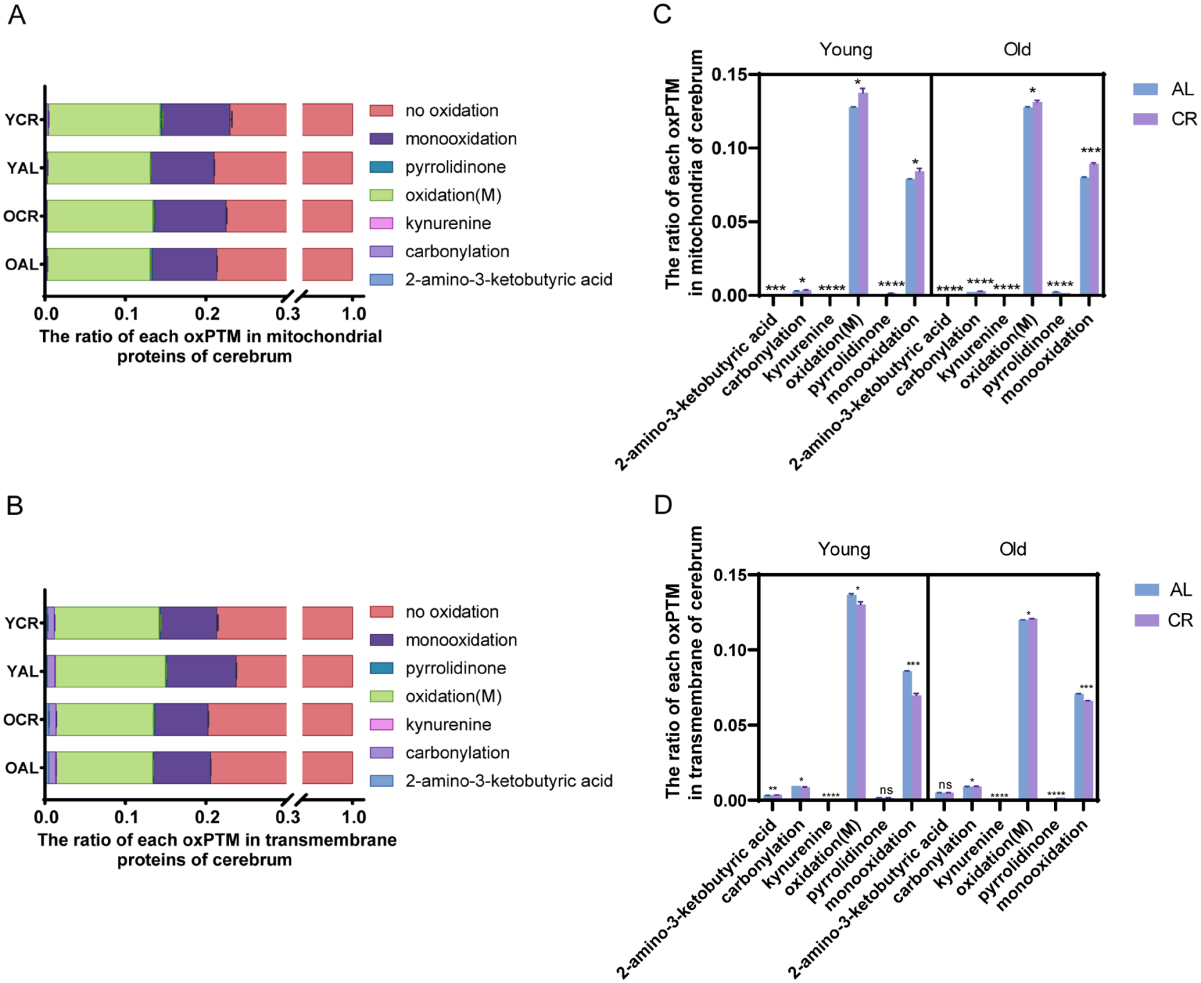
CR decreases oxidation(M) and monooxidation of TM proteins in young but increases oxidation(M) and monooxidation of mitochondrial proteins versus AL. The bar chart illustrates the ratio (modified peptides/all peptides) for each identified oxPTM in mitochondrial protein (A) and each identified oxPTM in transmembrane protein (B) of cerebrum in each animal (*n* = 3). The ratios were calculated as the ratio of the number of peptides with oxPTM to the total number of identified peptides. Error bars indicate the standard deviation among biological replicates (*n* = 3) per oxidative modification site. Effect of CR on each oxPTM in mitochondrial protein (C) transmembrane protein (D) of cerebrum in the short‐ and long‐term. *n* = 3, unpaired *t*‐test. *p < 0.05, **p < 0.01, ***p < 0.001, ****p < 0.0001, ns, not significant by Student *t*‐test.

One of the objectives of this study was to investigate whether and how particular oxPTMs are affected by age and by CR to estimate their relevance to the physiology of mitochondria in brain regions such as the cerebrum. The ratios of individual PTMs in each group were summed up and compared. It was found that the proportion of monooxidation and oxidation(M) were responsible for approximately 88% of the total oxPTMs (Table 2). In the context of mitochondrial proteins, monooxidation and oxidation(M) were identified as the predominant oxPTMs across all groups, thereby indicating that the overall sensitivity to ROS and CR intervention is mainly manifested by these two kinds of modification (Figure 5A). Regarding the impact of aging on individual oxPTMs, the effect was more prominent under AL than under CR, as reflected by the fact that 2‐amino‐3‐ketobutyric acid, kynurenine, pyrrolidinone, and monooxidation increased with aging (Figure S9A, Table 2). However, the most frequent oxidation(M) did not significantly change under AL, and under CR oxidation(M) and monooxidation displayed an opposite trend with a subtle decrease and increase, respectively. CR exerted a reducing effect on oxidation during aging, particularly the reduction of 2‐amino‐3‐ketobutyric acid, kynurenine, and oxidation(M), with oxidation(M) demonstrating a prominent change (Figure S9A, Table 2). Besides, the observed reduction in carbonylation and increase in pyrrolidinone and monooxidation in mitochondrial proteins of the cerebrum during aging occurred for both OAL and OCR, indicating that this phenomenon is independent of dietary influences during the aging process (Table S4, Table 2). Although the alterations in these three oxPTMs were consistent for both AL and CR, a more detailed analysis and calculation in Table 2 revealed that the effect was stronger under CR for carbonylation and monooxidation, whereas the enhancement for pyrrolidone was slightly reduced (Figure S9A, Table 2).

**TABLE 2:** Oxidative PTM alterations in the cerebrum during aging with an AL or CR diet.

Tissue	Diet	Age	PTMs	Mitochondrial proteins			Transmembrane proteins		
				Average ratio (%)	Diet effect	Age effect	Average ratio (%)	Diet effect	Age effect
Cerebrum	AL	Y	2‐amino‐3‐ketobutyric acid	0.06			0.33		
			Carbonylation	0.29			0.96		
			Kynurenine	0.00			0.06		
			Oxidation(M)	12.77			13.66		
			Pyrrolidinone	0.04			0.16		
			Monooxidation	7.88			8.60		
		O	2‐amino‐3‐ketobutyric acid	0.07		**↑**	0.51		**↑**
			Carbonylation	0.24		**↓**	0.88		**↓**
			Kynurenine	0.01		**↑**	0.06		**↑**
			Oxidation(M)	12.79		ns	11.99		**↓**
			Pyrrolidinone	0.25		**↑**	0.11		**↓**
			Monooxidation	8.00		**↑**	7.06		**↓**
	CR	Y	2‐amino‐3‐ketobutyric acid	0.09	**↑**		0.35	**↑**	
			Carbonylation	0.37	**↑**		0.87	**↓**	
			Kynurenine	0.01	**↑**		0.00	**↓**	
			Oxidation(M)	13.88	**↑**		13.00	**↓**	
			Pyrrolidinone	0.17	**↑**		0.15	ns	
			Monooxidation	8.50	**↑**		7.00	**↓**	
		O	2‐amino‐3‐ketobutyric acid	0.03	**↓**	**↓**	0.51	ns	**↑**
			Carbonylation	0.29	**↑**	**↓**	0.92	**↑**	**↑**
			Kynurenine	0.00	**↓**	**↓**	0.00	**↓**	—
			Oxidation(M)	13.11	**↑**	**↓**	12.07	**↑**	**↓**
			Pyrrolidinone	0.19	**↓**	**↑**	0.18	**↑**	**↑**
			Monooxidation	8.91	**↑**	**↑**	6.60	**↓**	**↓**

*Note:* The up arrow: significantly increased; The down arrow: significantly decreased; −: not available; Y: 6.5 months, O: 27 months. CR increases the amount of the two major oxPTMs monooxidation and methionine oxidation in mitochondrial proteins.

Abbreviations: AL, ad libitum; CR, calorie restriction; ns, not significant.

Strikingly, all oxPTM ratios were significantly increased in YCR compared with YAL (Table 2). In line with this trend, the ratio of oxPTMs, including carbonylation, oxidation(M), and monooxidation, continued to rise in OCR compared with OAL, whereas others presented a reduction (Figure 5C). Thus, CR increased the two most frequent oxPTMs in young and old animals. Moreover, the levels of oxPTMs, including 2‐amino‐3‐ketobutyric acid, kynurenine, and pyrrolidinone, were lower in the OCR group than in the OAL group. These results differ from the alterations observed in YCR compared with YAL. Additionally, the remaining oxPTMs had a higher ratio for the OCR samples compared with the OAL, but their relative increase was mostly less than that for YCR versus YAL.

In transmembrane proteins, oxidation(M) and monooxidation remained the most prevalent oxPTMs across all groups (Figure 5B). Regarding the role of aging, both PTMs decreased in TM proteins in young and old animals under the AL and CR diets, whereas for older animals, the CR diet differed less from the AL diet than that of younger animals (Figure S9B). The aging process in OCR resulted in an increase in carbonylation and pyrrolidinone levels compared with YCR. Conversely, a decrease in these levels was noted in OAL compared with YAL (Figure S9B, Table 2). The effect of diet was diametral for mitochondrial and TM proteins, whereas the latter exhibited a decrease in the majority and most frequent oxPTMs in young and old rats. In detail, the attenuation of oxPTMs, including carbonylation, kynurenine, oxidation(M), and monooxidation, was observed in YCR relative to YAL. Among these, kynurenine and monooxidation also demonstrated a reduction in OCR compared with OAL, although the effect of CR in lowering monooxidation ratio was weaker. Furthermore, the levels of carbonylation and oxidation(M) in OCR were elevated compared with those in OAL, which contrasts with the reduction observed when comparing YCR and YAL (Figure 5D, Table S4). In addition, the ratio of 2‐amino‐3‐ketobutyric acid was observed to increase exclusively in YCR compared with YAL, whereas pyrrolidinone demonstrated an increase in OCR compared with OAL (Figure 5D, Table 2).

## 3 Discussion

In our study, a comprehensive analysis of various protein oxidation types was performed to define their dependence on age, diet, and tissue. Notably, considerable variation in peptide detection between experimental groups was observed (Tables S5 and S6), reflecting biological proteome remodeling induced by aging and CR. Results for the heart were no significant change in oxidative modifications with age but a substantial decrease under CR (Figure 2). However, results for the cerebrum under CR were highly dissimilar (Figure 3), with an increase in mitochondrial protein oxidation and no significant effect of age, too. Of note, under all conditions, the relative oxidation level of proteins was approximately 40% lower in the cerebrum than in the heart (Figure S5). The use of proteomics enabled an independent and detailed analysis of several oxPTMs and revealed that methionine oxidation and monooxidation were most prominent under all conditions. In general, the obtained results demonstrate a complex relationship between CR, tissue, age, and oxidative PTM, which will be discussed in more detail below.

### 3.1 CR Drives Opposing, Tissue‐Specific oxPTM Trajectories in Heart and Cerebrum

Underlying reasons for the observed tissue‐specific pattern of oxPTMs could be differences in protein composition and abundance between heart and cerebrum (Calvo et al. 2016) and functional adaptation. Heart mitochondria should tolerate high ROS production as heart cells have been equipped with a highly sophisticated antioxidant scavenging system comprising antioxidant enzymes and antioxidants (Peoples et al. 2019). The brain also relies on aerobic metabolism to generate energy, yet it is more sensitive to oxidative stress and exhibits relatively low resistance (Cobley et al. 2018). Previously, slightly higher protein carbonylation was reported in the brain than in the heart (Sohal et al. 1994), whereas mitochondrial generation of H_2_O_2_ and superoxide was higher in the heart. Discrepancies of our study with this publication may be due to investigated tissue (cerebrum vs. total brain) and proteins (mitochondria vs. total) as well as the methods applied. Moreover, in our study bulk tissue analysis was done, even though heart and brain tissue contain different mitochondrial types, such as synaptic and non‐synaptic in cerebrum, or subsarcolemmal and the intermyofibrillar in heart. It can be speculated that on top of the inter‐tissue heterogeneity observed by us, intra‐tissue heterogeneity in oxPTMs exists. For heart, this idea is supported by observed differences in carbonylation of respiratory chain complexes (Padrao et al. 2011) and different changes in supercomplex composition during aging (Sandra Thilmany & Norbert A Dencher, unpublished results).

The tissue effects of CR on overall oxPTMs were diametrically opposed in our study. In both the short‐ and long‐term interventions, a notable decline in mitochondrial oxPTMs was observed in the heart in response to CR. Conversely, in the cerebrum, CR resulted in an unexpected elevation in mitochondrial oxPTMs (Figures 2A and 3A). The extent of alteration in mitochondrial oxPTMs also indicates that, at the tissue level, oxidative modification of proteins in the heart (19% relative decrease Y/O) is more susceptible to CR intervention than in the cerebrum (7% relative increase Y/O). In the analysis of how CR affects transmembrane proteins, a consistent and significant reduction of oxPTMs was observed in the heart (Figure 2B), which is consistent with a previous study by Sohal et al. (1994) indicating that the superoxide and hydrogen peroxide in mitochondria were both reduced by CR at either 9 or 23 months compared with the AL group. These findings are corroborated by the benefits presented in previous research, which indicated that CR delays cardiac aging in rats through apoptosis (Shirakabe et al. 2016), reverses the downregulated autophagy‐related proteins (Li et al. 2022), and reduces the rate of H_2_O_2_ release in mitochondria (Colom et al. 2007; Sohal et al. 1994). Contrary to the heart, in our study no consistent changes were identified in the cerebrum at the level of protein localization: CR induced a decrease in transmembrane protein oxidation but an increase in mitochondrial protein oxidation (Figure 3A,B). The perplexing observation of decreased transmembrane protein oxidation in the aged cerebrum, which was further reduced by CR, may be explained by the high turnover rate of membrane proteins in the brain (Rao et al. 2024). Additionally, two potential explanations could align this phenomenon with our results: either absolute oxidation is so low that the benefits of CR outweigh the molecular damage inflicted by ROS, or CR endows cells with improved oxidative and perhaps even general stress resistance according to the hormesis theory (Calabrese 2018; Dani et al. 2010; Masoro 2006). Indeed, the effect of CR on brain and neuronal cells is complex and comprises stimulation of mitochondrial activity (Levy et al. 2003) and biogenesis (Nisoli et al. 2005), and increased production of reactive nitrogen species (RNS) produced by NO synthase (Cerqueira et al. 2012). If one assumes that CR mainly elicited RNS production by NO synthase, it may explain the overall decrease in transmembrane protein oxidation after CR. In this situation, a major source of free radicals is not located anymore in the (mitochondrial) membrane but the cytoplasm. This tissue‐specific regulation was further exemplified by our protein‐level methionine oxidation analysis, which revealed that identical proteins can exhibit oxidation at distinct methionine residues with opposing responses to aging and CR between heart and cerebrum (Figure S8).

The idea of increased, yet tolerated free radical production under CR is corroborated by the study of Pifferi et al. (2018), in which it was illustrated that CR prolongs life in a primate model, yet simultaneously compromises the integrity of gray matter in the cerebrum without impairing cognitive performance. Thus, CR and free radicals play a multifaceted role in the brain by causing mild oxidative stress as well as triggering stress‐fighting signaling cascades, such as increased protein turnover.

### 3.2 Landscape of Major oxPTMs in Heart Versus Cerebrum

Oxidative protein modifications encompass a variety of covalent chemical alterations that can affect protein structure, stability, functionality, and interactions in both mitochondrial physiology and pathology during the aging process (Hamon et al. 2015). It is important to acknowledge that oxidative damage to proteins is a highly intricate process that results in the formation of a diverse array of products (Kehm et al. 2021). Among them, cysteine and methionine are particularly vulnerable to redox reactions because of the low oxidation potential of sulfur and its role in the regulation of physiological signaling pathways. Although cells possess ways to reverse some sulfur oxidations (Ugarte et al. 2010), the oxidative modification of many amino acids is irreversible and produces various kinds of oxidation products that, when not eliminated, can lead to pathological diseases. However, there is currently no clear and established correlation between specific oxPTMs and age‐related diseases. Importantly, it has been demonstrated that distinct tissues exhibit disparate biological components concerning their constituted structure, capacity for ROS defense (Limon‐Pacheco and Gonsebatt 2009), and intrinsic aging clock (Tian et al. 2023). With this in mind, we investigated the major oxidative modifications of mitochondrial proteins that occur during the aging process and the alterations resulting from CR intervention that enabled a comparison of relative amounts and trends among the various major oxPTMs in the heart and brain.

### 3.3 Divergent Trends for Individual oxPTMs in Mitochondrial Protein

Aging significantly increased monooxidation, one of the most abundant oxPTMs identified in our study, in mitochondrial proteins in the cerebrum but not in the heart (Figures S7A and S9A), further supporting the hypothesis that mitochondrial proteins in the cerebrum are more susceptible to ROS than those in the heart. This discrepancy may be attributed to differences in structure and function between two types of mitochondria, corroborated by recent research: La Padula et al. (2023) observed that heart mitochondria play a more prominent role in protecting against hypoxia than cortex mitochondria by reducing ROS production due to increased cytochrome oxidase activity. In addition, Giordano et al. (2011) revealed that the high level of paraoxonase 2 in the heart but its low abundance in the cortex indicates the low ability to retard oxidative stress, indicating a diminished capacity in the cerebrum to mitigate oxidative stress.

In our data, there was a considerable discrepancy in the alterations of individual oxPTMs between the heart and cerebrum mitochondrial protein following CR intervention (Tables 1 and 2). Moreover, divergent trends were observed in each tissue when comparing age and diet. This observation may be due to age at dietary intervention or duration of CR, and the observed divergent trends require further experimental investigation, because most previous studies on individual oxPTMs analyzed whole tissue and not the mitochondrial fraction, and experimental design (diet, age, etc.) varied. Even though a comparison of results is difficult and must be treated with caution, in the following an attempt is made: Concordant with the findings of a previous study (Cabiscol et al. 2014), our results also demonstrated that carbonylation increased significantly in both tissues under AL and CR with age (Tables S5 and S6). Nevertheless, there are clear variations in other oxPTMs between these two organs. The observed reduction in heart carbonylation levels in the comparison of YCR and YAL, as well as OCR and OAL, is concordant with the findings of Sohal et al. (1994). However, this is contrary to the brain protein carbonyl content in YCR compared to YAL, and OCR compared to OAL, which may be explained by the restriction to a crude mitochondrial fraction and the influence of region‐specific oxidations in organs, as illustrated by Granold et al. (2015), who proposed that protein oxidation is intimately associated with specific brain regions and species. In a study conducted by Pamplona, Portero‐Otin, Requena, et al. (2002), the levels of carbonylated proteins in heart mitochondria were evaluated using glutamic semialdehyde, and a notable decline in these levels was observed for animals that underwent CR treatment for 4 months. Similarly, it has been demonstrated that CR can diminish the level of cysteine oxidation in yeast, which increases with chronological age (Magherini et al. 2009). Obviously, the increased carbonylation observed in the cerebrum following CR treatment in our study differs from that reported in previous studies on the whole brain. Moreover, the varying duration of CR resulted in significantly distinct alterations in each oxPTM for the cerebrum, whereas the duration of CR administration in the heart did not significantly affect the trend of the majority of oxPTMs (Tables 1 and 2). However, a study (Judge et al. 2004) examining the effects of 2‐month CR intervention in 6‐month‐old rats observed an increase in protein carbonyls in heart mitochondria, which may have been caused by a reduction in the capacity for oxidant scavenging. These findings indicate the necessity for careful evaluation of various parameters that affect mitochondrial function and thereby protein oxidation.

### 3.4 No Uniform Trend for Individual oxPTMs in Transmembrane Proteins

Similar to mitochondrial proteins, there was no uniform trend for oxPTMs in transmembrane proteins; instead, they differed among tissues, diet, and age. Following the shift to CR diet, different roles of aging appear in the heart and cerebrum, manifested by contrasting alterations in 2‐amino‐3‐ketobutyric acid, carbonylation, and oxidation(M) (Figures S7B and S9B). In contrast, consistently decreased monooxidation and increased pyrrolidinone levels were observed in both tissues, independent of the influence of CR, but with age (Tables 1 and 2). Intriguingly, it has been reported that enzymatic proline oxidation in yeast mitochondria increases chronological lifespan, maintains mitochondrial membrane potential, and ATP production (Nishimura et al. 2021). Moreover, our data indicate that for OAL, compared with YAL, the majority of oxPTMs of transmembrane proteins in the heart remained unaltered with age (Figure S2B). This phenomenon agrees with Yan and Sohal (1998), who found that, out of all investigated proteins, only the carbonylation of one specific mitochondrial membrane protein progressed with age in the housefly model. Conversely, most oxPTMs in the cerebrum, except for increased 2‐amino‐3‐ketobutyric acid and kynurenine, exhibited a surprising decrease in OAL compared with YAL (Figure S3B). A previous study (Xiao et al. 2020) obtained similar results in that a considerable number of sites undergoing extensive cysteine oxidation in young tissues are specifically lost in aged tissues.

Unfortunately, there is a dearth of information about the role of specific oxPTMs in transmembrane proteins that could facilitate the elucidation of the molecular basis for CR in the heart and cerebrum. Importantly, the similar level of protection observed after both short‐ and long‐term CR suggests that its beneficial effects are not only rapidly inducible but also well‐sustained over time, particularly in the heart. It would appear that the benefits of CR are greater for young than for old animals in the cerebrum, as for the majority of oxidations, including carbonylation, oxidation(M), and pyrrolidinone, the relative decrease was more for YAL/YCR than OAL/OCR (Table 2). Conversely, long‐term CR may prove to be a more beneficial strategy for the heart transmembrane protein, as it has the potential to reduce carbonylation. Underlying mechanism supported by the findings of Li et al. (2022), could be upregulated autophagy triggered by long‐term CR, thereby improving mitochondrial quality in the heart.

### 3.5 Significance of Our Results for the Scope of the MFRTA Theory

The absence of a universal age‐related increase in oxPTMs in the mitochondrial subproteome aligns with observations in intact human skeletal muscle, which also shows no significant accumulation of global protein oxidation with age (Hutter et al. 2007). Our comprehensive, multi‐modification proteomic approach provides a new perspective on this longstanding debate. While our data are consistent with numerous previous reports (e.g., via immunoblotting) in showing a significant age‐dependent increase in protein carbonylation, a key prediction of the MFRTA, the integrated analysis of all oxidative modifications reveals a more complex picture. Specifically, we observed that the overall oxidative burden on mitochondrial proteins exhibited only a modest increase with age in the heart and no significant change in the cerebrum under AL and CR diet (Figure S5). This indicates that while carbonylation increases, other oxidative modifications follow distinct trajectories (e.g., some decrease or remain stable), resulting in a more balanced global profile than might be anticipated from studying carbonylation alone. Furthermore, and perhaps more strikingly, our analysis revealed that carbonylation levels in annotated mitochondrial or transmembrane proteins themselves predominantly decreased with age (Tables 1 and 2). This suggests the presence of intricate, selective regulatory mechanisms that mitigate oxidative damage in certain critical functional classes of proteins during aging, moving beyond the conventional narrative of a uniform age‐related increase. Notwithstanding the absence of a universal increase in oxPTMs, our study confirms a functional decline consistent with mitochondrial aging. Specifically, we observed a significant age‐related decrease in mitochondrial membrane fluidity (Figure S6), a parameter integral to membrane protein function and integrity. The process of rigidity is hypothesized to result primarily from lipid cross‐linking as a key contributor to reduced fluidity (Conte et al. 2015; Richter 1987). The restoration of membrane fluidity by long‐term CR directly links the dietary intervention to the preservation of this key functional property. Consequently, while the molecular footprint of aging (oxPTMs) is intricate and nonuniform, the functional outcome (membrane perturbance) is clear, and the protective role of CR on it is unequivocal, suggesting its benefits may operate through mechanisms beyond simply reducing global oxidative protein damage. Additionally, a previous study by some of us found that favorable redox balance is maintained even at advanced age, potentially playing a role in the scavenging and decomposition of ROS (Dani et al. 2010). Accordingly, it can be speculated that even without CR, most of ROS in mitochondria reacts with lipids and not proteins or can be scavenged and decomposed outside of the membrane, whereas under CR either less ROS occurs in the mitochondrial membrane or is eliminated by hydrophobic scavengers. MFRTA emphasizes the role of mitochondria in aging and proposes that the accumulation of oxidatively modified proteins contributes to mitochondrial dysfunction over time. Moreover, most studies have described CR as a decrease in ROS that is protective via various mechanisms (Kowaltowski 2011; Ungvari et al. 2008). Extensive research has been conducted on CR in various animal models, demonstrating that CR reduces ROS, protects mitochondrial structure, and improves mitochondrial function (e.g., (Guarente 2008) and (Demasi et al. 2021)). In the Drosophila model (Miwa et al. 2004), CR resulted in an extension of life span. In contrast, CR treatment did not alter ROS levels. In addition, the extent of oxidative damage to other biomolecules, including proteins and lipids, has been assessed to gain insight into the effects of aging and CR intervention. However, published studies (Bejma et al. 2000; Pamplona, Portero‐Otin, Bellmun, et al. 2002) on this topic have not yielded consistent results.

In our comprehensive analysis of mitochondrial oxidative protein modifications during the aging process that account for the effects of CR, it is important to note that the majority of the analyzed oxPTMs at advanced age do not adhere to the MFRTA, which postulates the accumulation of oxidative damage to proteins with age. However, even under conditions in which most oxPTMs increased or decreased, some oxPTMs frequently responded in the opposite direction to the global trend (Figures 4 and 5). Although previous studies often used other methods and investigated only one type of oxPTM, several also propose that the process of aging does not necessarily elicit uniform trends of oxidative protein modification within tissues or organisms (Dani et al. 2010; Xiao et al. 2020). This is corroborated by the reported diametrically opposite trends for the two oxidative stress biomarkers o‐tyrosine and methionine sulfoxide in aged liver mitochondria (Baldensperger et al. 2020) and the reduced oxidation of histones at higher age (Nakamura et al. 2010). A previous study by some of us (Rexroth et al. 2012) determined that the oxidation of ATP synthase was independent of age. Furthermore, Davies et al. (2001) compared the level of protein oxidative damage in the mitochondria of rat liver, brain, and heart tissue, assessed by the accumulation of protein carbonyls and other markers in total protein, and found there was no consistent pattern of increased oxidative damage in mitochondrial proteins with age. Our study did not reveal an outstanding relationship between aging or tissue and specific types of oxPTMs, which agrees with our previous work (Ramallo Guevara et al. 2016) that found no uniform trend for oxPTMs during the aging of the fungal model organism *Podospora anserina* using a similar proteomics approach. Collectively, these findings reflect the inherent complexity of the aging process. Nonetheless, our study offers a comprehensive analysis of protein oxidative modifications, providing a broader perspective than that afforded by the majority of existing research, which has predominantly investigated a limited range of oxPTMs, such as cysteine oxidation and carbonylation in one study. Furthermore, several reported findings (Hagopian et al. 2005; Judge et al. 2004; Xiao et al. 2020) have yielded conflicting alterations regarding different oxPTMs during the aging process that likely resulted from different experimental designs and aging models. Some of the intricate relationships between diverse oxPTMs and their influence on biological functions can be illuminated by the current study, which addresses several of the discrepancies observed in the literature by capturing the multifaceted nature of protein oxidation during aging. Given this context, it is not surprising that oxidative stress and altered oxPTMs are hallmarks of aging in certain circumstances, but not in others. It is now widely accepted that no single mechanism can fully account for the phenomenon of mitochondrial aging. Additionally, the beneficial effects of oxidized proteins in diverse physiological contexts were reviewed by Cai and Yan (2013). This suggests that not all oxPTMs and oxidized proteins have the same impact. When our results for the cerebrum are seen in light of previous studies, it can be assumed that the cerebrum has a lower buffer capacity for ROS and that CR causes mild oxidative stress, leading to the activation of protective signaling pathways.

## 4 Conclusion

In conclusion, it is evident that many of the obtained results are not in accordance with a simple oxidation‐accumulation theory of aging (MFRTA). Instead, it is of paramount importance to acknowledge that distinct tissues or organs and different proteins may exhibit disparate responses to age and to CR. Additionally, the modifications of each oxPTM are subject to the influences of aging, dietary patterns, the duration of dietary intervention, the specific tissue in question, and the genetic background, as demonstrated recently (Greenhill 2024; Li 2024) with these effects exhibiting considerable variability and frequent crosstalk. Furthermore, it must be noted that even when significant alterations were observed in our study, they were for the majority of conditions and PTMs relatively small. Lastly, it may even be speculated that the low amount of oxPTMs, particularly in the brain, and their slight increase under CR may be beneficial for aging according to the hormesis theory (Calabrese 2018; Dani et al. 2010; Masoro 2006).

### 4.1 Study Limitations and Future Directions

The present study is subject to the following limitations. Firstly, the limited sample size restricts the statistical power of proteomics comparisons. Secondly, while the employed approach identified global oxidation patterns, there was an absence of functional experiments. Thirdly, mitochondria were isolated from whole tissue without further separation procedures, for example, mitochondria from the cerebrum were not divided into synaptic and nonsynaptic subpopulations. Although this approach helps preserve mitochondrial integrity and improves yield, it does not allow differentiation of potential subset‐specific responses. Finally, the observations may be exclusive to the investigated rat strain and gender.

It is recommended that future studies place a greater emphasis on achieving large and diverse sample sizes, implementing targeted validation procedures, examining the effects of aging and CR on synaptic versus non‐synaptic mitochondria, and investigating other forms of oxidative damage, particularly to mtDNA, lipids, and glycans; this would provide a comprehensive picture and yield deeper mechanistic insights. Integrating morphological and functional evaluations with oxidative proteomic profiles in subsequent research would also help to bridge the gap between molecular modifications and tissue‐level physiological outcomes. The objective of these studies should be to establish the causal role of specific oxidative modifications in the process of aging, and to investigate the protective effects of CR.

## 5 Materials and Methods

### 5.1 Mitochondrial Samples From Different Rat Tissues and Design of Lifespan Extension Experiments

Male F344 Fischer rats of the species *Rattus norvegicus* were purchased from Charles River, Japan, and maintained under specific pathogen‐free (SPF) environment throughout the experiment at the Tokyo Metropolitan Institute of Gerontology (TMIG) with ethic approval number 10022. The tissue samples, heart and cerebrum, were obtained from the same animal whereby this study comprised four different conditions: two age groups and two nutrition groups. A total of 22 rats were used in the overall study, distributed across the groups as follows: young *ad libitum* fed rats (YAL, *n* = 5), young calorie restricted animals (YCR, *n* = 5), old *ad libitum* fed rats (OAL, *n* = 6), and old calorie restricted animals (OCR, *n* = 6). From these animals, comprehensive physiological data (e.g., body and organ weights) were collected. The cerebrum consisted of the brain regions cerebral cortex, basal ganglia and the limbic system, from which the hippocampus was separated. Samples were taken from rats that were either fed with free access to food every day (ad libitum) or were put on diet starting from week 6 after birth for either a short term (6.5 months) or a long‐term calorie restriction (27 months). Animals on calorie restriction (CR) were fed ad libitum (AL) on 3 days (Mondays, Wednesdays, and Fridays) per week (alternate day feeding, an alternate‐day fasting regimen) and free access to water every day so that the amount of food consumed was approximately 60% of the ad libitum fed levels. The composition of the diet was as follows: (per 100 g): 18.2 g protein, 4.8 g fat, 6.6 g mineral mixture, 5.0 g fiber, 57.9 g nitrogen‐free water‐soluble substance, and 7.5 g water. The caloric value of the diet was 348 kcal/100 g. The level of CR was calculated from the ratio of the body weight of CR animals and the body weight of AL nourished animals and was 40% for young animals and 37% for old animals. The experimental procedures were approved by the Animal Care and Use Committees of TMIG, Japan. The frozen tissues were transported to Germany in dry ice where crude mitochondrial fractions were prepared. For the subsequent mitochondrial proteomic analysis, a subset of three animals was randomly selected from each of the four groups (resulting in *n* = 3 per group for proteomics). The isolation of crude mitochondrial fractions was performed according to a previously established protocol as extensive purification of mitochondria may lead to the loss of interesting (e.g., senescent) mitochondria during harsh purification steps (Dani et al. 2010); thus, the conducted isolation of crude mitochondria preserved the delicate or impaired mitochondria. Briefly, tissues were homogenized in an ice‐cold buffer (50 mM Tris pH 7.4, 200 mM mannitol, 50 mM sucrose, 1 mM EDTA, and 5 mM EGTA) supplemented with antioxidants and protease inhibitors. The homogenate was then subjected to a series of differential centrifugation steps: 80 g for 10 min, 700 g for 10 min, and finally 10,000 g for 10 min to pellet the crude mitochondrial fraction. The resulting pellet was washed and resuspended in a storage buffer (50 mM Tris, 200 mM mannitol, 50 mM sucrose, plus protease inhibitor cocktail). Importantly, the metal chelation and reducing agents were employed to avoid artifactual oxidation of the samples. Mitochondria from both heart and cerebrum tissues were isolated using an identical purification protocol to ensure methodological consistency and to enable direct comparison of mitochondrial parameters across tissues. Subsequently, from the proteome data only proteins with mitochondria as annotated subcellular localization were retained for further analysis.

### 5.2 OxyBlot Analysis

Protein carbonylation was analyzed using the OxyBlot Kit (Chemicon S7150). Briefly, sample aliquots were derivatized with DNPH to label carbonyl groups, while control aliquots received the derivatization‐control solution. A positive control was generated by metal‐catalyzed oxidation of BSA. Derivatized proteins were separated by SDS‐PAGE and transferred to a PVDF membrane activated with 95% ethanol. The membrane was blocked with 1% BSA in PBS‐T, then incubated overnight at 4°C with the primary anti‐DNP antibody provided in the kit. Detection was performed using an HRP‐conjugated secondary antibody and chemiluminescence.

### 5.3 Measurement of Mitochondrial Membrane Fluidity

Mitochondrial membrane fluidity was assessed by fluorescence anisotropy using the hydrophobic probe 1,6‐diphenyl‐1,3,5‐hexatriene (DPH). This parameter reports on both the order parameter and the dynamics of DPH, which directly reflect the viscosity of the lipid bilayer. The physical principles and experimental validation of DPH fluorescence anisotropy measurements are detailed by Heyn, Cherry, and Dencher (1981). Briefly, the isolated mitochondria from rat cerebrum were incubated with 10 μM DPH in PBS for 1 h in the dark. Steady‐state fluorescence anisotropy was measured at 37°C using a PTI M III fluorescence spectrometer (excitation 376 nm, emission 455 nm). The anisotropy value (r) was calculated, where a decrease in r indicates an increase in membrane fluidity and vice versa.

### 5.4 Protein Digestion and Peptide iTRAQ‐Labeling

Processing of mitochondrial samples using a modified in‐filter protein digestion (FASP) procedure and tryptic digestion was performed according to Ramallo Guevara et al. (2016). All mitochondrial samples from each tissue sample were mixed in equal proportions to create an iTRAQ‐labeled mix that served as a spike‐in internal standard. A 4‐plex iTRAQ kit was used for relative quantification and therefore three biological replicates of each condition were labeled with the remaining iTRAQ reagents as described previously (Ramallo Guevara et al. 2016).

### 5.5 Mass Spectrometry

Samples were resuspended in 2% ACN in 0.1% FA to a final concentration of 1.5 μg/μL by sonication for 10 min before one‐dimensional nLC‐ESI‐MS/MS analysis. The measurements were performed on an LTQ‐Orbitrap Elite mass spectrometer coupled to a nanoACQUITY gradient UPLC pump system. The peptides were separated in the same manner using reversed‐phase chromatography as described in Ramallo Guevara et al. (2016). However, 5 μL of the sample was loaded directly onto the 25‐cm analytical column using the nanoACQUITY autosampler, the column oven was set to 55°C, and the re‐equilibration of the column was extended by 20 min. Data‐dependent acquisition on the LTQ‐Orbitrap Elite was operated via instrument method files of Xcalibur (Rev. 2.1.0) in positive ion mode at a spray voltage of 1.8 kV. Methods using exclusively HCD fragmentation when acquiring MS/MS spectra consisted of an Orbitrap full MS scan followed by up to 20 HCD‐Orbitrap MS/MS spectra (Top20, Bottom20; explanation see Ramallo Guevara et al. (2016)) on the most abundant ions detected in the full MS scan. The full MS scan was performed in the Orbitrap in the range of 150–2000 m/z at a resolution of 120,000. The AGC for full MS experiments was set to 1 × 10^6^ with a maximum ion injection time of 500 ms. For peptide identification and reporter ion quantification, HCD fragmentation spectra were acquired with a normalized collision energy of 35%, isolation width of 1.5th, and activation time of 0.1 ms at a resolution of 15,000. The AGC for the MS/MS experiments was set to 1 × 10^4^ at a maximum ion accumulation time of 250 ms. Dynamic exclusion was enabled with a repeat count of one and a 45 s exclusion duration window. Unassigned charge states and singly charged ions were rejected from MS/MS. For each 4‐plex sample, 4 technical replicates were performed, that is, 2 times Top20 and 2 times Bottom20.

### 5.6 MS Data Processing

Protein identification and quantification in this iTRAQ experiment was performed with MaxQuant software (v2.1.3.0 for 1st and 2nd search in cerebrum and 1st search in heart; v2.3.0.0 for 2nd search in heart) using the implemented Andromeda search algorithm (Cox et al. 2011). All acquired RAW files from one tissue were processed in one database search using MaxQuant software and were searched against *the Rattus norvegicus* protein database downloaded from http://www.uniprot.org (UP000002494; 2022/08/17) with 37,919 protein sequence entries, complemented by a database of common protein contaminants according to the standard Andromeda settings. Searches were performed using trypsin and LysC specificity allowing two missed cleavages. Peptides with at least 6 amino acids were considered for identification while allowing LysC N‐terminal cleavage to proline. Identification across different replicates was achieved by enabling the matching between runs option in MaxQuant within a time window of 0.7 min. The precursor‐ion mass tolerance was set to 20 ppm for the first search and 4.5 ppm for main search. The fragment ion mass tolerance for HCD‐MS/MS spectra was kept by default of 20 ppm. The false discovery rate, determined by searching a reversed database, was set at 0.01 for both peptides and proteins. The analysis type in MaxQuant was set to Reporter ion MS2, and 4‐plex iTRAQ was selected with a reporter mass tolerance of 0.01 Da. Filtering by precursor intensity fraction of > 0.75 was enabled to avoid unwanted quantification of co‐isolated peptides, and the identification of second peptides was disabled. Acetylation of the protein N‐terminus and pyro‐glutamic acid at peptide N‐terminal glutamine residues were set as variable modifications. Additionally, oxidative modifications were considered as variable modification sites and were quantified consecutively in two database searches as indicated in Table S1. The minimum Andromeda score values for MS/MS identification of modified peptides were maintained by default (Min. score: 40; Min delta score: 6). Site localization probabilities were determined by MaxQuant using the PTM scoring algorithm, as described by Olsen et al. (2006) and Cox and Mann (2008).

### 5.7 Data Analysis

The reporter intensity count of each group (YAL, OAL, YCR, and OCR) for each oxidative peptide modification type was summarized and divided by the total peptide count for each sample in the heart and cerebrum to obtain the relative ratio for comparison (ratio is used below). This normalization to identified peptides was performed to account for technical variance (digestion efficiency, sample load, etc.). Given that the obtained mitochondrial fraction was not highly purified and soluble, as well as membrane proteins were obtained, filtering of the results based on protein annotation was envisioned for detailed data analysis. For this purpose, transmembrane proteins were filtered using the keywords “transmembrane”, and acknowledged mitochondrial proteins were filtered using the gene ontology cellular component (GOCC) with terms containing “mitochondri” respectively, after the addition of the UniProt annotation of *Rattus norvegicus* in Perseus (v2.0.10.0) (Tyanova et al. 2016). The statistical analyses were conducted using Prism version 9.0 (GraphPad Software). The results were expressed as the mean ± standard deviation. The impact of dietary regimen (AL or CR) and age (young or old) on outcomes in the multiple groups was evaluated using two‐way ANOVA. Significant interactions between CR and age in two‐way ANOVA were followed by the assessment of main effects using Bonferroni's correction for multiple comparisons. For individual PTMs, the significance of the difference between two groups was evaluated using a two‐tailed Student's *t*‐test. The results were considered statistically significant at *p* < 0.05.

## Author Contributions

A.P. and N.A.D. designed the experiments; N.A.D., S.G., M.D.S., and S.Y. were involved in sample generation and characterization; C.R.‐G. performed the proteomics experiments; M.F. performed the OxyBlot assay; Data were analyzed by S.F. and A.P.; S.F. wrote this manuscript; N.A.D., S.F., A.P., and S.G. revised the manuscript.

## Funding

This work was supported by Provincial Special Project Grant, Foreign Experts Program, Long‐Term Foreign Experts Program (9202‐0210227525) of Jiangxi province, China and German Federal Ministry for Education and Research (BMBF) (FKZ0315584).

## Conflicts of Interest

The authors declare no conflicts of interest.

## Supporting information


**Appendix S1:** acel70339‐sup‐0001‐AppendixS1.docx.

## Data Availability

The proteomics RAW data and MQ results are available in the iProX repository (IPX0013383002) at https://www.iprox.cn/. The number of modified and unmodified peptides for each sample is in Appendix S1 of this article.
