# Supplementary material for: Not Aging but Calorie Restriction Strongly Affects Protein Oxidation in Heart and Brain Mitochondria
Source: Aging Cell. 2025 Dec 28;25(1):e70339. doi: 10.1111/acel.70339 (PMC12745344; doi:10.1111/acel.70339)
Supplement: Supplementary file 1 — Appendix S1: acel70339‐sup‐0001‐AppendixS1.docx. [file ACEL-25-e70339-s001.docx]

**Supporting information**

**Figure S1.** Protein carbonylation in cortex mitochondria decreases with age. Representative OxyBlot (right) and corresponding total protein stain (left) of mitochondrial proteins from young (Y1, Y2) and aged (O1, O2) rats. Standard (BSA), negative (non-derivatized sample), and positive (H₂O₂-treated) controls are shown.

**Figure S2.** Survival curves. The number of rats at the start of the study was 5-6 in each group. AL: Ad libitum, CR: Calorie restriction based on dietary restriction (an alternate-day fasting strategy).

**Figure S3.** Average body weight. Average of the body weight of the individual animals. Error bars are standard error of means from n=5 animals for YAL and YCR and n=6 for OAL and OCR. AL: Ad libitum, CR: calorie restriction, Y: 6.5 months, O: 27 months. Two-way ANOVA. *P<0.05, **P<0.01, ***P<0.001, ****P<0.0001.

**Figure S4.** Average and relative organ weights of heart and cerebrum. (A, B) Average absolute wet weight of the (A) heart and (B) cerebrum for each experimental group. (C, D) The relative weight of the (C) heart and (D) cerebrum, expressed as a percentage of individual body weight. Error bars are standard error of means from n=5 animals for YAL and YCR and n=6 for OAL and OCR. AL: Ad libitum, CR: calorie restriction, Y: 6.5 months, O: 27 months. Two-way ANOVA. *P<0.05, **P<0.01, ***P<0.001, ****P<0.0001, ns: not significant.

**Figure S5.** Global alterations in oxidative PTMs in the heart and cerebrum based on all identified proteins (proteins annotated as mitochondrial and others). Total ratio of oxPTMs in heart (A) and oxPTMs in cerebrum (B). Histograms represent the mean ± SD. n = 3; two-way ANOVA. The ratios were calculated as the number of identified peptides with oxPTM normalized to the total number of identified peptides. AL: Ad libitum, CR: calorie restriction, Y: 6.5 months, O: 27 months, oxPTMs: oxidative protein modifications. *P<0.05, **P<0.01, ***P<0.001, ****P<0.0001, ns: not significant.

**Figure S6.** Mitochondrial membrane fluidity assessment. Steady-state fluorescence anisotropy of the membrane probe 1,6-diphenyl-1,3,5-hexatriene (DPH) was measured, which is inversely related to membrane "fluidity." Data are presented as the mean of technical replicates (n=10). Error bars representing the standard error. AL: Ad libitum, CR: calorie restriction, Y: 6.5 months, O: 27 months, oxPTMs: oxidative protein modifications. Significance: *p < 0.05, **p < 0.01. Black asterisks denote changes induced by aging (YAL vs. OAL; YCR vs. OCR); red asterisks denote changes induced by CR (YAL vs. YCR; OAL vs. OCR).

**Figure S7.** The effect of aging in the heart under AL and CR modes. (A) Each oxPTM in mitochondrial proteins. (B) Each oxPTM in transmembrane proteins. Histograms represent the mean± SD. n=3, unpaired t-test. *P<0.05, **P<0.01, ***P<0.001, ****P<0.0001, ns: not significant. The most frequent oxPTMs were not significantly affected by age in the AL group.

**Figure S8.** Tissue-specific methionine oxidation patterns in mitochondrial proteins. (A) Heart and (B) cerebrum methionine oxidation profiles across experimental groups (OAL, OCR, YAL, YCR). Each row represents an individual oxidation site (Protein_MetPosition) in mitochondrial proteins that showed significant changes (p < 0.05) in at least one comparison. Colors indicate relative oxidation levels (red: increased; blue: decreased) after protein-level normalization. Data demonstrate tissue-specific and mitochondrial proteins to oxidative modifications during aging and dietary intervention. AL: Ad libitum, CR: calorie restriction, Y: 6.5 months, O: 27 months.

**Figure S9.** The effect of aging in the cerebrum under the AL and CR modes as the ratio oxPTM (modified peptides/all peptides). (A) Each oxPTM in mitochondrial proteins. (B) Each oxPTM in transmembrane proteins. Histograms represent the mean± SD. n=3, unpaired t-test. *P<0.05, **P<0.01, ***P<0.001, ****P<0.0001, ns: not significant. The most frequent oxPTMs oxidation(M) and monooxidation decreased in TM proteins in young and old animals fed the AL or CR diet.

Table S1. The mass difference between the amino acid and its oxidation product was considered as a variable modification during the MaxQuant database search.

| **Modification designation** | **Examination of amino acids** | **Database search** | **Monoisotopic mass shift (Da)** |
| --- | --- | --- | --- |
| Oxidation | Lys, Asp, His, Leu, Met, Phe, Pro, Trp | First | +15.99492 |
| Pyrrolidinone | Pro |  | -30.01057 |
| Carbonylation | Arg, Leu, Pro |  | +13.97927 |
| Kynurenine | Trp | Second | +3.99490 |
| 2-amino-3-ketobutyric acid | Thr |  | -2.10560 |

Table S2. Proportions of filtered annotated mitochondrial and transmembrane proteins in heart and cerebrum.

| **Tissue** | **Diet** | **Age** | **Mitochondrial proteins** | **Transmembrane proteins** |
| --- | --- | --- | --- | --- |
| Heart | AL | Y | 44.86 | 17.94 |
|  |  |  | 44.59 | 17.88 |
|  |  |  | 44.87 | 17.73 |
|  |  | O | 43.68 | 19.69 |
|  |  |  | 43.52 | 19.50 |
|  |  |  | 43.58 | 19.21 |
|  | CR | Y | 39.76 | 18.74 |
|  |  |  | 39.85 | 18.86 |
|  |  |  | 39.94 | 18.75 |
|  |  | O | 38.81 | 18.73 |
|  |  |  | 38.91 | 18.74 |
|  |  |  | 38.79 | 18.79 |
| Cerebrum | AL | Y | 24.57 | 25.00 |
|  |  |  | 24.58 | 24.96 |
|  |  |  | 24.55 | 24.93 |
|  |  | O | 24.41 | 25.81 |
|  |  |  | 24.37 | 25.77 |
|  |  |  | 24.38 | 25.78 |
|  | CR | Y | 24.82 | 25.58 |
|  |  |  | 24.77 | 25.61 |
|  |  |  | 24.81 | 25.65 |
|  |  | O | 25.38 | 25.96 |
|  |  |  | 25.35 | 25.97 |
|  |  |  | 25.37 | 25.95 |

| **Tissue** | **Diet** | | **Age** | | **Mitochondrial proteins** | | **Transmembrane proteins** | |  |
| --- | --- | --- | --- | --- | --- | --- | --- | --- | --- |
| Heart | | AL | | Y | | 44.86 | | 17.94 | |
|  |  |  |  |  |  | 44.59 | | 17.88 | |
|  |  |  |  |  |  | 44.87 | | 17.73 | |
|  |  |  |  | O | | 43.68 | | 19.69 | |
|  |  |  |  |  |  | 43.52 | | 19.5 | |
|  |  |  |  |  |  | 43.58 | | 19.21 | |
|  |  | CR | | Y | | 39.76 | | 18.74 | |
|  |  |  |  |  |  | 39.85 | | 18.86 | |
|  |  |  |  |  |  | 39.94 | | 18.75 | |
|  |  |  |  | O | | 38.81 | | 18.73 | |
|  |  |  |  |  |  | 38.91 | | 18.74 | |
|  |  |  |  |  |  | 38.79 | | 18.79 | |
| Cerebrum | | AL | | Y | | 24.57 | | 25 | |
|  |  |  |  |  |  | 24.58 | | 24.96 | |
|  |  |  |  |  |  | 24.55 | | 24.93 | |
|  |  |  |  | O | | 24.41 | | 25.81 | |
|  |  |  |  |  |  | 24.37 | | 25.77 | |
|  |  |  |  |  |  | 24.38 | | 25.78 | |
|  |  | CR | | Y | | 24.82 | | 25.58 | |
|  |  |  |  |  |  | 24.77 | | 25.61 | |
|  |  |  |  |  |  | 24.81 | | 25.65 | |
|  |  |  |  | O | | 25.38 | | 25.96 | |
|  |  |  |  |  |  | 25.35 | | 25.97 | |
|  |  |  |  |  |  | 25.37 | | 25.95 | |

The ratio (%) is calculated as the number of annotated mitochondrial proteins or transmembrane proteins divided by the number of all identified proteins, respectively. AL: Ad libitum, CR: calorie restriction, Y: 6.5 months, O: 27 months.

**Table S3.** Ratios of identified carbonylated peptides in heart from mitochondrial proteins or transmembrane proteins.

| **Tissue** | **Diet** | **Age** | **Mitochondrial proteins** | **Transmembrane proteins** |
| --- | --- | --- | --- | --- |
| Heart | AL | Y | 0.41 | 0.57 |
|  |  |  | 0.39 | 0.55 |
|  |  |  | 0.53 | 0.75 |
|  |  | O | 0.48 | 0.51 |
|  |  |  | 0.48 | 0.69 |
|  |  |  | 0.57 | 0.82 |
|  | CR | Y | 0.09 | 0.84 |
|  |  |  | 0.09 | 0.86 |
|  |  |  | 0.09 | 0.85 |
|  |  | O | 0.01 | 0.22 |
|  |  |  | 0.01 | 0.22 |
|  |  |  | 0.01 | 0.10 |

The ratio (%) is calculated as the number of identified carbonylated peptides divided by the number of all identified peptides. AL: Ad libitum, CR: calorie restriction, Y: 6.5 months, O: 27 months.

**Table S4.** Ratios of identified carbonylated peptides in cerebrum from mitochondrial proteins or transmembrane proteins.

| **Tissue** | **Diet** | **Age** | **Mitochondrial proteins** | **Transmembrane proteins** |
| --- | --- | --- | --- | --- |
| Cerebrum | AL | Y | 0.30 | 0.96 |
|  |  |  | 0.30 | 0.96 |
|  |  |  | 0.26 | 0.95 |
|  |  | O | 0.24 | 0.89 |
|  |  |  | 0.24 | 0.85 |
|  |  |  | 0.24 | 0.90 |
|  | CR | Y | 0.37 | 0.84 |
|  |  |  | 0.37 | 0.86 |
|  |  |  | 0.39 | 0.90 |
|  |  | O | 0.29 | 0.92 |
|  |  |  | 0.28 | 0.92 |
|  |  |  | 0.29 | 0.93 |

The ratio (%) is calculated as the number of identified carbonylated proteins divided by the number of all identified proteins, respectively. AL: Ad libitum, CR: calorie restriction, Y: 6.5 months, O: 27 months.

**Table S5.** Number of identified carbonylated peptides in the heart

| **Tissue** | **Diet** | **Age** | **Number of identified carbonylated peptides** | **The number of background peptides** | **Ratio (%)** |
| --- | --- | --- | --- | --- | --- |
| Heart | AL | Y | 202 | 11554 | 1.75 |
|  |  |  | 210 | 12066 | 1.74 |
|  |  |  | 177 | 9387 | 1.89 |
|  |  | O | 201 | 9675 | 2.08 |
|  |  |  | 204 | 9627 | 2.12 |
|  |  |  | 183 | 8274 | 2.21 |
|  | CR | Y | 151 | 20425 | 0.74 |
|  |  |  | 163 | 20885 | 0.78 |
|  |  |  | 164 | 21035 | 0.78 |
|  |  | O | 132 | 15554 | 0.85 |
|  |  |  | 130 | 15643 | 0.83 |
|  |  |  | 145 | 15928 | 0.91 |

AL: Ad libitum, CR: calorie restriction, Y: 6.5 months, O: 27 months. The background peptides represent the total number of identified peptides. The number of identified carbonylated peptides was divided by the number of background peptides.

**Table. S6** The number of identified carbonylated peptides in the cerebrum.

| **Tissue** | **Diet** | **Age** | **Number of identified carbonylated peptides** | **Number of background peptides** | **Ratio (%)** |
| --- | --- | --- | --- | --- | --- |
| Cerebrum | AL | Y | 272 | 24610 | 1.11 |
|  |  |  | 272 | 24618 | 1.10 |
|  |  |  | 265 | 24766 | 1.07 |
|  |  | O | 434 | 30876 | 1.41 |
|  |  |  | 432 | 31063 | 1.39 |
|  |  |  | 437 | 31011 | 1.41 |
|  | CR | Y | 374 | 28067 | 1.33 |
|  |  |  | 378 | 27643 | 1.37 |
|  |  |  | 353 | 26605 | 1.33 |
|  |  | O | 473 | 27139 | 1.74 |
|  |  |  | 481 | 27510 | 1.75 |
|  |  |  | 471 | 27141 | 1.74 |

AL: Ad libitum, CR: calorie restriction, Y: 6.5 months, O: 27 months. The background peptides are the total identified peptides; the ratio is calculated as the number of identified carbonylated peptides divided by the number of background peptides.

Supplementary compressed datasets

Dataset S1. ITRAQ proteome data of each oxPTM in heart. (Dataset S1.xlsx)

Dataset S2. ITRAQ proteome data of each oxPTM in cerebrum. (Dataset S2.xlsx)
